# Unravelling pain in diabetic neuropathy patients: Exploring the relationship between perceived pain severity, lifestyle, and coping strategies mediated by self-focused attention and rumination: A cross-sectional study

**DOI:** 10.1016/j.heliyon.2025.e42397

**Published:** 2025-01-31

**Authors:** Fatemeh Hosseini, Amirhossein Yavari, Arya Haddadi

**Affiliations:** a Department of Clinical Psychology, Faculty of Medical Sciences, Islamic Azad University, Hamedan Branch, Hamedan, Iran; b Behavioral Disorders and Substance Abuse Research Center, Hamadan University of Medical Sciences, Hamadan, Iran; c Clinical Psychology, Faculty of Medical Sciences, Hamedan Branch, Islamic Azad University, Hamedan, Iran

**Keywords:** Severity of pain perception, Lifestyle, Pain coping strategies, Self-focused attention, Rumination, Diabetic neuropathy

## Abstract

**Objective:**

Diabetes ranks highly among the world's non-communicable diseases, bringing about a host of physical and psychological impacts on those it afflicts. One such complication is neuropathy, often resulting in significant pain and discomfort. Diabetic neuropathy patients' perceived pain intensity, lifestyle, and pain management strategies measures will be examined in this study. This study also analyses how self-focused attention and rumination mediate this dynamic in Hamedan, Iran, 2023 patients.

**Methods:**

The study population comprised neuropathy patients in Hamadan province, Iran. A sample of 253 individuals was selected for the study. Data collection involved several questionnaires: The McGill Pain Questionnaire, Lifestyle Questionnaire, the Coping Strategies Questionnaire, the Self-Focused Attention Questionnaire, and the Rumination Questionnaire. The research model was evaluated using structural equation modeling with AMOS software version 24.

**Results:**

Path analysis revealed that the model exploring the relationship between lifestyle-based severity of pain perception and pain coping strategies, mediated by self-focused attention and rumination, was a good fit for patients with diabetic neuropathy. (P < 0.05)

**Conclusion:**

The findings suggest that an improved lifestyle and more effective pain management strategies are linked to decreased severity of pain perception in patients with diabetic neuropathy. Additionally, the research indicates that self-focused attention and high levels of rumination may lead to increased severity of pain perception in these individuals. This research has the potential to advance the understanding of diabetic neuropathy's underlying mechanisms and enhance treatment approaches, ultimately playing a significant role in improving the quality of life for these patients.

## Highlights

• Adopting suitable lifestyle changes can alleviate pain perception and enhance coping.
• Self-focused attention and rumination play crucial roles in mediating pain severity.
• Understanding these mechanisms can enhance treatment and quality of life.

## 1 Introduction

Diabetes, A chronic metabolic disorder characterized by high blood sugar levels due to insufficient insulin production or utilization, has become a significant non-communicable disease globally, primarily due to lifestyle changes and the rising prevalence of diabetes [1], with estimates suggesting that over 592 million people will be affected by 2035. Alarmingly, many individuals with diabetes remain undiagnosed. Additionally, the widespread lack of awareness about diabetes and its management contributes to severe health complications and, tragically, loss of life [2]. Diabetes, with its potential for causing long-term health issues and disabilities, is a serious concern.

Neuropathy, a chronic complication affecting 50–90 % of those with type 1 and type 2 diabetes, often leads to diabetic foot ulcers [3]. Symptoms of neuropathy include intense pain, hypersensitivity, muscle weakness and fatigue, burning sensations, and leg discomfort akin to electric shocks. These debilitating symptoms can significantly impair a person's ability to perform daily activities [4]. Considering the physical and psychological consequences that this disease brings, today, it is believed that to treat and control the various implications of various diseases, in addition to biological and physiological issues, psychological and social factors should also be addressed [5]. One of the biological and psychological factors that should be paid special attention to in patients is the severity of pain perception.

Pain is defined as an unpleasant sensory experience associated with actual or potential tissue damage [6]. Numerous factors, such as biological, psychological, and social aspects, impact pain intensity. Pain encompasses both sensory and emotional dimensions. The sensory element addresses the perceived intensity of pain, while the emotional facet examines the degree of distress a person endures [7].

Severity of pain perception is a significant factor that hampers an individual's ability to perform daily tasks and engage socially, significantly impacting various aspects of life [7]. The biopsychosocial model suggests that the severity of pain experienced by patients can be anticipated based on the interaction of biological, psychological, and social factors [8].

Lifestyle, a significant factor influencing pain intensity is defined as the habits, behaviors, and daily routines related to diet, exercise, and overall health management [9], encompasses the distinct ways individuals, groups, and societies live, incorporating a variety of tangible and intangible factors [9]. The unhealthy lifestyle choices made by individuals can worsen their health conditions and possibly result in their demise. According to the World Health Organization (WHO), roughly half of all deaths caused by different illnesses are attributed to unhealthy habits. Therefore, adopting a suitable lifestyle is crucial for managing diseases and enhancing overall quality of life [10].

Seemingly, in addition to lifestyle, other psychological factors, such as coping strategies, have a relationship with severity of pain perception [11]. Coping strategies are the cognitive and behavioral techniques individuals use to manage stress or difficult situations [11]. Pain coping strategies have two categories: proactive and reactive strategies. Proactive strategies involve engaging in activities despite the pain, redirecting focus from the pain, and practicing muscle relaxation techniques. On the other hand, reactive strategies involve negative behaviors like catastrophic thinking, reliance on others for help, and reducing one's daily routines due to pain. It is a multi-faceted approach that requires a combination of efforts [11]. The strategies used by people to cope with pain affect their ability to perceive pain, control pain, endure pain, and continue doing daily tasks. Using reactive strategies causes patients to experience more severe pain, anxiety, excessive worry, physical disability, and mental disorders, such as depression [11].

Correspondingly, some factors can act as mediating factors. One of these factors is self-focused attention, indicating that people who focus and pay considerable attention to their pain, in addition to experiencing more pain, have fewer resources to cope with pain [12]. Self-focused attention is the tendency to focus one's attention inward, reflecting on personal thoughts and emotions [12]. Self-focused attention may stem from memories of past emotions, attitudes, or events that individuals have encountered, influencing their current feelings, emotions, and behaviors [13]. Self-focused attention may intensify and maintain negative emotions such as anxiety and can also heighten the sensation of pain in individuals with chronic conditions [12].

Other mediating factors include rumination, which refers to the repetitive and passive fixation on negative thoughts, often centered around past distress or problems. This process can impact mental health as individuals become preoccupied with self-critical, negative reflections on personal issues [14]. While the intent may be to alleviate negative emotions, this behavior often leads to a deepening of depressive feelings [14]. Prolonged rumination poses a significant risk to mental well-being as it can exacerbate or prolong depressive episodes. It can also impair cognitive functions, hinder emotional processing, and result in social withdrawal [15]. Donatti et al. (2017) investigated the relationship between positive coping strategies and depression, stress, and pelvic pain. They found a positive correlation between positive coping strategies and less pain experience [16]. Furthermore, Vertsberger et al. (2023), in a group of patients who suffered from back pain, found that rumination affects their severity of pain perception and lifestyle [17].

The present research team could not find a study that investigated the relationship between the severity of pain perception based on lifestyle and pain coping strategies in patients with diabetic neuropathy. Currently, considering the limited research in this field in Iran and the increasing number of people who have diabetes and its complications and adverse effects, this research is focused on investigating the relationship between lifestyle and pain coping strategies, the severity of pain perception, and the mediating role of self-focused attention and rumination in patients with diabetic neuropathy.

## 2 Materials and methods

### 2.1 Statistical population and sample

The present study is a cross-sectional correlational analysis focusing on individuals with diabetic neuropathy residing in Hamadan, Iran, in 2023. According to Tabachnick and Fidell (2007), the minimum sample size for modeling studies should be at least 110 participants [18]. Two hundred fifty-three individuals with diabetes from Hamadan were selected to secure an adequate sample size for this research through a convenience sampling method to serve as a study sample.

### 2.2 Procedure

According to the hours of the patients attending the Social Security Hospital and Mahdieh Clinic in Hamedan, a trained clinical psychologist was present and distributed and completed the questionnaires among the volunteer patients. The study's objectives and all relevant explanations were provided to the participants. Inclusion criteria consisted of having diabetes and neuropathy, a willingness to participate in the study, and not currently taking psychiatric medications. Exclusion criteria included incomplete or distorted questionnaires and a willingness to withdraw from the study for any reason.

### 2.3 Bias

Possible biases may involve self-reporting bias. To mitigate this, we ensured the presence of a trained psychologist during the completion of the questionnaires.

### 2.4 Ethical consideration

All ethical guidelines for the research were adhered to in accordance with the Declaration of Helsinki.The permission to conduct the research was obtained from the Ethics Committee in Biomedical Research of the Islamic Azad University, Hamedan branch, Iran, with the code IR.IAU.H.REC.1402.085.

### 2.5 Research tools

1. *The McGill Pain Questionnaire* designed by Melzack In 1975 [19], which assessed pain symptoms in twenty individuals on a scale from one to ten. The study confirmed the tool's validity and reliability. Analyses revealed four dimensions of pain: sensory, emotional, evaluative, and diverse experiences. In 2016, Behbahani et al. investigated the questionnaire's psychometric properties within an Iranian context, establishing that the Persian adaptation maintained strong validity and reliability with a Cronbach's alpha coefficient exceeding 0.8 [20].
2. *The Lifestyle Questionnaire* developed by Kern et al., In 1993, to assess personal lifestyle [21]. This questionnaire has 62 questions and includes five primary subscales: Belonging-interest, coping, responsibility, need for approval, and being cautious based on a five-point Likert scale with items such as “When I was a child,” “When a task I was doing it right,” and “I liked to be noticed,” to measure lifestyle attributes. The options are scored in such a way that a score of 1 is taken for completely disagree to a score of 5 for completely agree, and 15 questions are scored inversely. In the study of Akbari et al., the validity and reliability of the Persian version of this questionnaire were confirmed with a Cronbach's alpha coefficient calculated to be above 0.7 [22].
3. *The Pain Coping Strategies Questionnaire* developed by Rosenstile and Keefe in 1983 [23], comprising 42 items aimed at assessing different pain management tactics such as diverting attention, reinterpreting pain sensations, using positive self-talk, ignoring pain, catastrophizing, and resorting to prayer or hope. Respondents are prompted to read each statement and rate the extent to which they employ each strategy when experiencing pain on a 7-point scale ranging from zero to six. Each strategy is scored from 0 to 36, with higher scores indicating a greater reliance on that particular coping mechanism [23]. The study by Seydi et al. confirmed the reliability and validity of the Persian translation of the questionnaire, reporting Cronbach's alpha coefficients for six cognitive strategies (attention diversion 0.75, reinterpretation of pain 0.74, self-talk 0.78, ignoring pain 0.77, catastrophizing and praying 0.75, and hopefulness 0.81) and one behavioral strategy (increased behavioral activity 0.72) [24].
4. *The Self-Focused Attention or Focus of Attention Questionnaire*, developed by Woody et al., In 1977, assessing the attention focus of individuals with social anxiety during social interactions. It features two 5-item subscales: one for self-focused attention and another for external attention [25]. Each item is graded on a 5-point scale from “not at all true” to “completely true.” The score of each subscale can be calculated and used separately. Bakhtiaripoor et al. confirmed the validity and reliability of this questionnaire in their study, reporting Cronbach's alpha coefficients of 0.75 for self-focused attention and 0.86 for external focus of attention [26].
5. *The Rumination Questionnaire* was designed by Treynor et al., In 1993, evaluating four different types of reactions to negative mood [27]. This questionnaire has 22 statements, and the respondents are asked to rate each on a scale from 1 (never) to 5 (often). Based on empirical evidence, the scale of rumination responses has high internal reliability. In their research, Bagherzadeh et al. confirmed the validity and reliability of this questionnaire, with a Cronbach's alpha coefficient of 0.89 indicating strong reliability [28].

### 2.6 Statistical analysis

Data were analyzed using descriptive methods, such as frequency tables and graphs, and inferential statistics including correlation and regression analyses in SPSS-26 and path analysis in AMOS-24 were used to examine the data and test hypotheses.

## 3 Results

Table 1 summarizes the demographic characteristics of the participants, including age, gender, education, marital status, employment, and awareness of disease duration.

**Table 1:** Demographic characteristics of the participants.

Demographic Category	Subcategory	Number of Participants	Percentage (%)
**Age Group**	Under 30	62	24.5
	30 to 45	79	31.2
	Over 45	–	31.2
	**Average Age**	–	36.92
**Gender**	Male	78	30.8
	Female	175	69.2
**Education Level**	No Diploma	86	34.0
	Diploma	73	28.9
	Post-Diploma	11	4.3
	Bachelor's Degree	48	19.0
	Master's Degree or Higher	35	13.8
**Marital Status**	Single	62	24.5
	Married	172	68.0
	Divorced	19	7.5
**Employment Status**	Housewives	82	32.4
	Employees	134	53.0
	Self-employed	25	9.9
	Other	12	4.7
**Awareness of Disease Duration**	Less than 3 years	35	13.8
	4–6 years	79	31.3
	7–10 years	56	22.1
	More than 10 years	83	32.8

The results of the lifestyle, pain coping strategies, self-focused attention, rumination, and intensity of pain perception variables analysis were as follows:

Table 2 shows the Cronbach's alpha coefficients of each variable and the research variables' mean and standard deviation. As can be seen, Cronbach's alpha coefficients of all components and variables are close to or higher than 0.7. Based on this, the dialogues of each questionnaire used to measure the variables of the current research had an acceptable internal consistency. Furthermore, the investigations showed that the correlation between the variables was in the expected direction and in line with the theories of the research field.

**Table 2:** Mean, standard deviation, and Cronbach's alpha coefficient of variables.

Variables	Mean	SD	Cronbach's Alpha	95 % Confidence Interval
Lifestyle- Belonging/social interest	13.57	3.26	0.66	(13.17, 13.97)
Lifestyle–coping	22.17	5.42	0.79	(21.50, 22.84)
Lifestyle–responsibility	19.80	4.92	0.71	(19.19, 20.41)
Lifestyle–need for approval	14.32	3.07	0.64	(13.94, 14.70)
Lifestyle–being cautious	11.68	2.96	0.63	(11.32, 12.04)
Coping strategy–Returning attention	13.69	4.12	0.81	(13.18, 14.20)
Coping strategy–reinterpretation	12.23	3.71	0.79	(11.77, 12.69)
Coping strategy–catastrophizing	14.48	4.36	0.75	(13.94, 15.02)
Coping strategy–ignoring	13.51	4.03	0.80	(13.01, 14.01)
Coping strategy–prayer and hope	18.33	5.12	0.83	(17.70, 18.96)
Coping strategy–self-talk	16.75	4.68	0.78	(16.17, 17.33)
Self-focused attention	15.93	3.12	0.74	(15.55, 16.31)
Rumination	67.04	11.51	0.91	(65.62, 68.46)
Intensity of pain perception–sensory perception	58.47	9.76	0.94	(57.27, 59.67)
Intensity of pain perception–emotional perception	34.97	7.23	0.77	(34.08, 35.86)
Intensity of pain perception–pain assessment	7.17	2.75	–	(6.83, 7.51)
Intensity of pain perception–various pains	32.90	7.94	0.73	(31.92, 33.88)

All the research assumptions were established, including the normality of univariate data, collinearity, normality of multivariate data, and uniformity of dispersion among the data.

### 3.1 Fit indices for the measurement model

Table 3 reveals that, except for the comparative fit index (CFI), the remaining indices derived from confirmatory factor analysis failed to indicate an adequate fit between the measurement model and the gathered data. Consequently, the fit indices improved by introducing three covariances among the error terms related to strategies for pain management, pre-correction, and post-correction. As depicted in Table 3, these modifications suggest that the measurement model is now appropriately aligned with the data collected.

**Table 3:** Model fit indices.

Fit Indices Model	Initial Measurement Model	Revised Measurement Model	Cutoff Point^a^
Chi-Square^a^	307.98	220.92	–
Degrees of Freedom	87	84	–
df^b^/^2^χ	3.54	2.63	Less than 3
GFI^c^	0.897	0.918	0.90 <
AGFI^d^	0.843	0.872	0.850 <
CFI^e^	0.915	0.947	0.90 <
RMSEA^f^	0.101	0.080	0.08 <

a -Chi-Square

b - normed chi-square.

c -Goodness Fit Index.

d -Adjusted Goodness Fit Index.

e -Comparative Fit Index.

f -Root Mean Square Error of Approximation.

### 3.2 Standards factor loadings of measurement model indicators

**Fig. 1:**
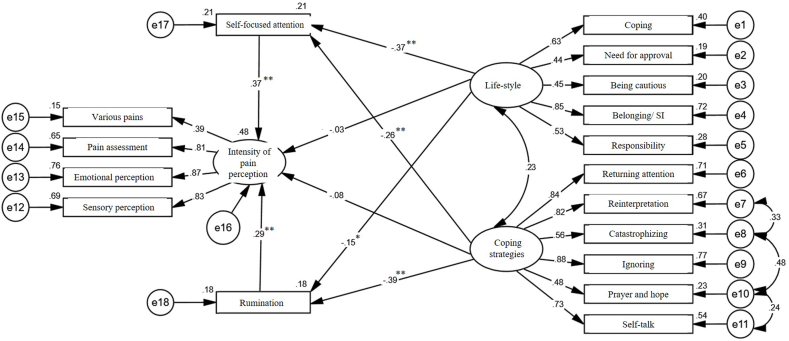
Research structural model utilizing standardized data.

Table 4 highlights that the highest factor loading is associated with the indicator for neglect (β = 0.891), while the lowest loading corresponds to the indicator for various pains (β = 0.356). Given that all indicators’ factor loadings exceed 0.32, they possess adequate strength to assess the variables in this study. Importantly, as Tabachnick and Fidel stated in 2007, factor loadings are deemed excellent if they are 0.71 or higher, very good between 0.63 and 0.70, good between 0.55 and 0.62, moderately good from 0.45 to 0.55, low between 0.32 and 0.44, and weak if below 0.32. Fig. 1 presents the measurement model of the research and its corresponding factor loadings based on standardized data.

**Table 4:** Model parameters of measurement model in confirmatory factor analysis.

Current Variable - Indicator	β	T
Lifestyle–Belonging/social interest	0.887	0.001
Lifestyle–coping	0.618	0.001
Lifestyle–responsibility	0.516	0.001
Lifestyle–need for approval	0.424	0.001
Lifestyle–being cautious	0.425	0.001
Coping strategy–Returning attention	0.834	0.001
Coping strategy–reinterpretation	0.818	0.001
Coping strategy–catastrophizing	0.544	0.001
Coping strategy–ignoring	0.891	0.001
Coping strategy–prayer and hope	0.455	0.001
Coping strategy–self-talk	0.725	0.001
Intensity of pain perception–sensory perception	0.809	0.001
Intensity of pain perception–emotional perception	0.890	0.001
Intensity of pain perception–pain assessment	0.808	0.001
Intensity of pain perception–various pains	0.356	0.001

### 3.3 Structural modeling

As Table 5 shows, all the fit indices obtained from structural equation modeling (SEM) analysis support the fit of the research structural model with the collected data. Thus, it was concluded that the structural model of the research fits the collected data.

**Table 5:** Structural model fit indices.

Fit Indices	Structural Model
Chi-Square	270.32
Degrees of Freedom	109
df/^2^χ	2.48
GFI	0.913
AGFI	0.866
CFI	0.939
RMSEA	0.076

Table 6 shows the path coefficients between the variables in the structural model of the research. The total path coefficient between lifestyle and severity of pain perception is negative and significant (p = 0.001, β = 0.212). Therefore, it was concluded that the lifestyle of patients with diabetic neuropathy has a negative and significant relationship with the severity of pain perception. The total path coefficient between pain coping strategies and pain severity is negative and significant (p = 0.001, β = 0.290). Accordingly, it was concluded that pain coping strategies in patients with diabetic neuropathy have a negative and significant relationship with the severity of pain perception. The path coefficient between self-focused attention and pain severity is positive and significant (p = 0.001, β = 0.369). Besides, it was concluded that in patients with diabetic neuropathy, self-focused attention significantly correlates with the severity of pain perception. The statistical path analysis revealed a positive and noteworthy relationship between rumination and pain severity, with a p-value of 0.001 and a path coefficient (β) of 0.287. This suggests that increased rumination among these patients is associated with a more significant severity of pain perception. The use of Baron and Kenny's model showed that the path coefficient between lifestyle and pain severity through self-focused attention is negative and statistically significant (p = 0.001, β = 0.137). It was concluded that self-focused attention significantly and negatively mediates the relationship between lifestyle and the severity of pain perception in patients with diabetic neuropathy. Additionally, Baron and Kenny's model revealed that the path coefficient linking pain coping strategies to the intensity of pain perception via self-focused attention is negatively significant (p = 0.001, β = −0.096). Consequently, this suggests that self-focused attention serves as a significant negative mediator in the association between pain coping strategies and the severity of pain perception in patients with diabetic neuropathy. Applying Baron and Kenny's method revealed that the path coefficient linking lifestyle to the severity of pain perception via rumination is negative and statistically significant (p = 0.001, β = −0.113). This indicates that rumination serves as a negative and significant mediator in the connection between lifestyle and severity of pain perception among patients with diabetic neuropathy. Similarly, the path coefficient between pain coping strategies and severity of pain perception, mediated by rumination, is statistically significant and negative (p = 0.038, β = −0.044). This suggests that rumination also negatively and significantly mediates the relationship between patients' pain coping strategies and their severity of pain perception in diabetic neuropathy.

**Table 6:** Total, direct, and indirect path coefficients between research variables in the structural model.

Path	Current Variables	B	S.E	Β	P	95 % Confidence Interval
**Direct**	Coping strategy → Rumination	−0.628	0.101	−0.394	0.001	(-0.592, −0.196)
	Coping strategy →Self-focused attention	−0.226	0.055	−0.255	0.001	(-0.363, −0.147)
	Coping strategy → pain perception	−0.151	0.164	−0.083	0.393	(-0.404, 0.238)
	Lifestyle → Rumination	−0.929	0.492	−0.152	0.044	(-1.116, 0.812)
	Lifestyle → Self-focused attention	−1.263	0.290	−0.373	0.001	(-0.941, 0.195)
	Lifestyle → pain perception	−0.219	0.557	−0.031	0.658	(-1.123, 1.061)
	Rumination → pain perception	0.327	0.081	0.287	0.001	(0.128, 0.446)
	Self-focused attention → pain perception	0.760	0.174	0.369	0.001	(0.028, 0.71)
**Indirect**	Coping strategy → pain perception	−0.0377	0.084	−0.207	0.001	(-0.372, −0.042)
	Lifestyle → pain perception	−1.263	0.413	−0.181	0.001	(-0.99, 0.628)
**Total**	Coping strategy → pain perception	−0.528	0.153	−0.290	0.001	(-0.59, 0.01)
	Lifestyle → pain perception	−1.482	0.318	−0.212	0.001	(-0.835, 0.411)

As the figure below shows, the sum of squared multiple correlations (R2) for the severity of pain perception variable was equal to 0.48, indicating that rumination, self-focused attention, pain coping strategies, and lifestyle explain a total of 48 % of the variance of the severity of pain perception in patients with diabetic neuropathy.

## 4 Discussion

The overall findings from the study suggest that the exploration into how the severity of pain perception, influenced by lifestyle and pain coping strategies, mediated by self-focused attention and rumination, relate to each other in patients with diabetic neuropathy showed an adequate fit. In the following, the obtained results are discussed in detail.

People respond to life's traumas, such as illness, in various ways. Living with chronic conditions brings significant alterations to an individual's daily routine. Nevertheless, some individuals with chronic illnesses, such as diabetes, resist modifying their lifestyles. This resistance leads to increased disease complications that can severely affect patients' specific goals and everyday tasks. Adopting a healthy lifestyle and appropriate pain coping strategies are among the essential factors that help people face problems such as pain caused by illness [29]. Using appropriate strategies to deal with pain or crises caused by the disease, in addition to the positive effects it has in controlling or reducing the complications of the disease, also protects patients from suffering from many psychological problems [30].

Utilizing ineffective coping strategies can lead to an inability to manage emotions and external circumstances, resulting in persistent negative thoughts and exacerbating their pain perception. When a person's focus deviates from its normal state, their sense of self-efficacy and control over their surroundings and internal emotions diminishes, which, in turn, affects cognitive biases [30]. Accordingly, individuals in a state of self-focused attention, especially those suffering from chronic pain, may feel ill-equipped to control the intensity of their pain. As a result, they often perceive themselves as falling short of their own standards, which may further elevate their pain severity [30].

When the severity of pain perception exceeds an individual's tolerance, the body is placed in a state of distress and avoidance. Pain is often perceived as an indicator of potential harm, triggering stress and anxiety. Anxiety, in turn, contributes significantly to rumination, and together, these elements intensify the individual's experience of pain [31].

The lifestyle of patients with diabetic neuropathy was found to have a significant negative relationship with the severity of pain perception, consistent with the findings of Zhang et al. [10] and Delgado-Velandia et al. [32]. Individuals with diabetic neuropathy who lead high-stress lifestyles, smoke, or engage in poor dietary habits may experience increased pain severity [33]. To mitigate this, individuals with chronic illnesses must adopt lifestyle adjustments and employ effective management strategies. Failure to do so can disrupt multiple aspects of life, diminishing cognitive function and personal relationships [34].

Similarly, pain coping strategies in patients with diabetic neuropathy were negatively and significantly related to the severity of pain perception. These findings align with those of Nasika et al. [35] and Kuzu et al. [36]. Pain, a universal human experience, is primarily influenced by how individuals cope with it. Ineffective strategies, such as avoidance, where people deny or trivialize the consequences of a crisis, can lead to maladaptive behaviors like alcohol consumption or reliance on sedatives [36,37]. These behaviors not only impair mood but also worsen pain severity, making it harder to manage.

Self-focused attention in patients with diabetic neuropathy was positively and significantly related to pain intensity. This finding was consistent with Stimmel et al. [12] and Diotaiuti et al. [38]. Self-focused attention, a cognitive bias, plays a central role in many disorders, amplifying negative emotions and chronic emotional experiences. Excessive self-absorption can exacerbate pathological conditions, such as depression [13]. When individuals are overly focused on their internal state, they neglect external cues, worsening their ability to solve problems and cope with life challenges, including chronic pain [38].

Rumination, similarly, had a significant positive relationship with pain perception in diabetic neuropathy patients. This finding supports the work of Schütze et al. [39] and Hannibal et al. [40]. Patients frequently express concerns about their illness, often leading to rumination, which exacerbates psychological disorders and other negative consequences of the disease. Persistent negative thoughts can disrupt problem-solving abilities, leading to an overestimation of pain, particularly when patients ruminate on their condition instead of seeking solutions.

Self-focused attention negatively mediated the relationship between lifestyle and pain perception severity in patients with diabetic neuropathy. Although no previous literature specifically addresses this relationship, it can be interpreted through the lens of lifestyle's impact on cognitive processes. Individuals who adopt maladaptive lifestyles may become more self-focused, honing in on their perceived inadequacies, leading to a diminished sense of control over their pain, which exacerbates their discomfort. This heightened self-focus also impacts mental health, further amplifying the severity of pain.

Similarly, self-focused attention negatively mediated the relationship between pain coping strategies and pain perception severity. When individuals excessively focus on their internal state and the negative aspects of their condition, they may experience psychological distress, disrupting their ability to cope with pain. This cognitive pattern hinders their ability to process external information that might help alleviate their pain or challenge their negative thoughts.

Rumination also significantly mediated the relationship between lifestyle and pain perception severity in diabetic neuropathy patients. Although no direct studies explore this relationship, it can be explained through the role of lifestyle in shaping cognitive biases. Negative rumination can dominate one's thinking, leading to a hyper-focus on the disease and its symptoms. As a result, these recurring thoughts heighten emotional distress, exacerbating pain and diminishing problem-solving capabilities [17,41].

Lastly, rumination negatively mediated the relationship between pain coping strategies and pain perception severity. Ineffective coping strategies, especially when paired with persistent negative thinking, leave individuals more vulnerable to rumination, further decreasing their confidence in managing their illness. This cycle leads to greater psychological suffering, reinforcing both rumination and pain perception [39].

### 4.1 Limitations and suggestion

Despite the innovation of this research and the valuable information it provides, the study faced several limitations, such as the non-cooperation of some internal medicine and endocrinology clinics in distributing the questionnaire among referring patients. Furthermore, the worsening health of some of the referring patients hindered their ability to participate. Moreover, the study's reliance solely on self-report questionnaires as the data collection method may have introduced a single-method bias into the findings.

Considering the use of self-report questionnaires in this research, it is suggested that other tools, such as interviews, be used for measurement in future research. Proposedly, this research will be conducted in other societies with other demographic characteristics to ensure the generalizability of the results obtained to society.

### 4.2 Clinical implications

The study's findings highlight the importance of addressing lifestyle factors, pain coping strategies, and psychological elements like self-focused attention and rumination in managing pain for diabetic neuropathy patients. Clinically, encouraging healthier lifestyle choices and teaching adaptive coping mechanisms can significantly reduce pain perception. Additionally, psychological interventions like CBT or ACT could help manage negative thought patterns, improving both pain control and mental well-being. A holistic, multidisciplinary approach combining these strategies may enhance patient outcomes and improve their quality of life.

## 5 Conclusions (4–5 lines–optional)

The present study concluded that lifestyle and pain coping strategies were related to the severity of pain perception, and self-focused attention and rumination played a mediating role. The relationship between these factors is significant in controlling the pain of patients with diabetes. Following a suitable lifestyle among patients can affect a large part of their problems related to pain control, rumination, and even self-focused attention. Furthermore, using appropriate pain coping skills among patients increases the amount of control over the complications caused by the disease and empowers people to control their internal and external conditions. According to the discussed issues, this research can contribute to progress in understanding the mechanisms of diabetic neuropathy, improve the treatment strategies of these patients, and play an essential role in improving their quality of life. Based on the obtained results, addressing psychological issues such as self-focused attention and rumination can significantly reduce the severity of the pain experienced by patients. Although time-consuming, lifestyle changes and new coping strategies can effectively lessen pain by improving the mediating variables identified in this research.

## CRediT authorship contribution statement

**Fatemeh Hosseini:** Writing – review & editing, Writing – original draft, Resources, Investigation, Conceptualization. **Amirhossein Yavari:** Writing – review & editing, Supervision, Formal analysis, Data curation. **Arya Haddadi:** Writing – review & editing, Writing – original draft, Supervision, Project administration, Methodology, Conceptualization.

## Ethical approval statement

The aims of the research were explained to the participants, and their informed consent was then obtained. This study was performed according to the principles expressed in the Declaration of Helsinki and was approved by the Deputy of the Research and Ethics Committee of Islamic Azad University of Hamedan (ID: IR.IAU.H.REC.1402.085).

## Data availability statement

Not applicable.

## Declaration of generative AI and AI-assisted technologies in the writing process

During the preparation of this work the author(s) used [ChatGPT4/Grammarly] in order to help translate the manuscript from Persian to English and improve the language. After using this tool/service, the author(s) reviewed and edited the content as needed and take(s) full responsibility for the content of the publication.

## Funding

This research received no specific grant from any funding agency in the public, commercial, or not-for-profit sectors.

## Declaration of competing interest

The authors declare that they have no known competing financial interests or personal relationships that could have appeared to influence the work reported in this paper.
