# Supplementary material for: Unravelling pain in diabetic neuropathy patients: Exploring the relationship between perceived pain severity, lifestyle, and coping strategies mediated by self-focused attention and rumination: A cross-sectional study
Source: Heliyon. 2025 Jan 31;11(3):e42397. doi: 10.1016/j.heliyon.2025.e42397 (PMC11848071; doi:10.1016/j.heliyon.2025.e42397)
Supplement: Multimedia component 1 [file mmc1.doc]

**به نام خدا**

**معرفی پرسشنامه**

**پرسشنامه استاندارد سبک زندگی کرن و همکاران 1993 (**BASIS-A**)**

پرسشنامه سبک زندگی توسط کرن و همکاران در سال (1993) به منظور سنجش سبک زندگی طراحی و تدوین شده است. این پرسشنامه دارای 62 سوال و شامل پنج خرده مقیاس اصلی تعلق – علاقه؛ کنار آمدن؛ مسئولیت پذیری؛ نیاز به تایید و محتاط بودن و پنج خرده مقیاس فرعی حمایتی سخت گیری؛ مستحق بودن؛ مورد علاقه ی همه بودن؛ کوشش برای رسیدن به کمال و ملایمت می باشد و بر اساس طیف پنج گزینه ای لیکرت با سوالاتی مانند (وقتی من بچه بودم، هنگامی که کاری را درست انجام می دادم دوست داشتم مورد توجه قرار گیرم) به سنجش سبک زندگی می پردازد.

**تعریف عملیاتی متغیر پرسشنامه**

در این تحقیق منظور از سبک زندگی نمره ای است که پاسخ دهندگان به سوالات 62 گویه ای پرسشنامه سبک زندگی می دهند.

**مولفه های پرسشنامه و پرسشنامه**

این پرسشنامه دارای 5 خرده مقیاس اصلی شامل هفت گویه برای مشخص کردن تعلق – علاقه اجتماعی ( عبارات شماره ی 1، 6، 21،22،29 ،34 ،54 و 10گویه کنار آمدن ( عبارات شماره 17، 26، 30، 35، 40،44،49 ،52 ،53 ،59) هشت گویه مسئولیت پذیری ( عبارات شماره 3، 8، 13، 18، 23، 27، 32، 36، ) هفت گویه نیاز به تایید ( عبارات شماره ی 9، 11، 14، 16، 19، 24، 28، )و پنج گویه محتاط بودن ( عبارات شماره ی 5، 10، 12، 15، 20) و پنج خرده مقیاس فرعی حمایتی که عبارتند از شش گویه سخت گیری ( عبارت شماره ی 4، 25، 31، 32، 39، 61، ) شش گویه مستحق بودن ( عبارت شماره ی 2، 7، 42، 43، 48، 58، ) سه گویه مورد علاقه ی همه بودن ( عبارات شماره 41، 51، 56) پنج گویه برای مشخص کردن کوشش برای رسیدن به کمال ( عبارات شماره ی 37، 38، 46، 47، 57) ملایمت (عبارات شماره ی 50، 45*،* 55، 60، 62) می باشد *.*

| **کاملا**  **موافق** | **موافق** | **بی نظر** | **مخالف** | **کاملا مخالف** | وقتی من بچه بودم . . . . . . . . . . . . . . . . . . . . | ردیف |
| --- | --- | --- | --- | --- | --- | --- |
|  |  |  |  |  | از بازی با بچه های دیگر لذت می بردم | 1 |
|  |  |  |  |  | مورد توجه ویژه بودم | 2 |
|  |  |  |  |  | دوست داشتم به دیگران بگویم چه انجام دهند . | 3 |
|  |  |  |  |  | بارها در مدرسه احساساتم جریحه دار می شد. | 4 |
|  |  |  |  |  | نمی توانستم با والدینم صادق باشم . | 5 |
|  |  |  |  |  | در زمینه های متعدد اعتماد به نفس داشتم | 6 |
|  |  |  |  |  | بیشتر از آنچه حقم بود داشتم . | 7 |
|  |  |  |  |  | بچه های دیگر را امر ونهی می کردم | 8 |
|  |  |  |  |  | چندین دوست نزدیک داشتم | 9 |
|  |  |  |  |  | یکی از والدینم احساس می کرد من به درد نخور هستم | 10 |
|  |  |  |  |  | به دیگران احساس تعلق می کردم | 11 |
|  |  |  |  |  | مشکلات زیادی برای والدینم به وجود می آوردم | 12 |
|  |  |  |  |  | احساس می کردم خیلی قدرتمند هستم | 13 |
|  |  |  |  |  | دوست داشتم به بهترین نحو کارهای محوله را انجام دهم | 14 |
|  |  |  |  |  | در خانواده احساس بی کفایتی می کردم | 15 |
|  |  |  |  |  | با یک گروه به خوبی جور بودم | 16 |
|  |  |  |  |  | موقع تنبیه با دیگران تسویه حساب می کردم | 17 |
|  |  |  |  |  | احساس رییس بودن می کردم | 18 |
|  |  |  |  |  | وقتی در کاری موفق می شدم احساس مهم بودن می کردم | 19 |
|  |  |  |  |  | یکی از والدینم برای مثال پدر یا مادرم از دستم عصبانی بود | 20 |
|  |  |  |  |  | مورد قبول بچه های دیگر بودم | 21 |
|  |  |  |  |  | با دیگران رفتار دوستانه ای داشتم | 22 |
|  |  |  |  |  | می خواستم بچه های دیگر را کنترل کنم | 23 |
|  |  |  |  |  | هنگامی که کاری را درست انجام می دادم دوست داشتم مورد توجه قرار گیرم | 24 |
|  |  |  |  |  | این که رهبرباشم یا دنباله رو برایم فرقی نمی کرد | 25 |
|  |  |  |  |  | اگر به هدفم نمی رسیدم عصبانی می شدم | 26 |
|  |  |  |  |  | به خوبی نشان می دادم که چه کسی رییس است | 27 |
|  |  |  |  |  | وقتی در مدرسه خوب بودم احساس مورد قبول بودن می کردم | 28 |
|  |  |  |  |  | معاشرتی بودم | 29 |
|  |  |  |  |  | با بچه های دیگر زیاد دعوا می کردم | 30 |
|  |  |  |  |  | می خواستم در فعالیت های مدرسه مسولیتی داشته باشم | 31 |
|  |  |  |  |  | طوری رفتار می کردم که مورد توجه قرار بگیرم | 32 |
|  |  |  |  |  | از والدینم می ترسیدم | 33 |
|  |  |  |  |  | از اینکه با بچه های دیگر بودم احساس لذت می کردم | 34 |
|  |  |  |  |  | بعد از تنبیه لجبازتر می شدم | 35 |
|  |  |  |  |  | وقتی بچه های دیگر به حرفم عمل می کرند احساس مهم بودن می کردم | 36 |
|  |  |  |  |  | دوست داشتم بزرگتر ها را بیشتر خوشحال کنم وموجب ناراحتیآنها نشوم | 37 |
|  |  |  |  |  | به نظر نمی آمد بتوانم کار درستی در خانه انجام بدهم | 38 |
|  |  |  |  |  | دوست داشتم در کار های گروهی شرکت داشته باشم | 39 |
|  |  |  |  |  | وقتی دیگران مرا اذیت می کردند با آنها دعوا می کردم | 40 |
|  |  |  |  |  | نسبت به این که ایا بزرگتر ها با انچه من انجام می دادم موافق بودن نگران بودم | 41 |
|  |  |  |  |  | دوست نداشتم مسولیتی داشته باشم | 42 |
|  |  |  |  |  | در خانه ناز پرورده بودم | 43 |
|  |  |  |  |  | دوست داشتم تلافی کنم | 44 |
|  |  |  |  |  | در دوست پیداکردن مشکل داشتم | 45 |
|  |  |  |  |  | از اینکه می توانستم والدینم را خوشحال کنم شاد می شدم | 46 |
|  |  |  |  |  | در بیشتر کارهای که ازمایش وکوشش می کردم موفق می شدم | 47 |
|  |  |  |  |  | در خانه هر چیزی که می خواستم به من داده می شد | 48 |
|  |  |  |  |  | به طور علنی تلاش می کردم که با دیگران تصفیه حساب کنم | 49 |
|  |  |  |  |  | می خواستم تنها باشم | 50 |
|  |  |  |  |  | به تایید یکی از والدینم نیاز داشتم | 51 |
|  |  |  |  |  | سعی می کردم از انجام دادن کارها ی خانه خودداری کنم | 52 |
|  |  |  |  |  | را ه خودم را پیش می گرفتم | 53 |
|  |  |  |  |  | برای اینکه جز گروه بمانم سعی می کردم محکم وقوی باشم | 54 |
|  |  |  |  |  | احساس می کردم که قربانی عصبانیت دیگران هستم | 55 |
|  |  |  |  |  | برایم مهم نبود که مرا دوست داشته باشند | 56 |
|  |  |  |  |  | کارهای بسیاری را درست انجام می دادم | 57 |
|  |  |  |  |  | لوس بودم | 58 |
|  |  |  |  |  | انتقام می گرفتم | 59 |
|  |  |  |  |  | همیشه می خواستم سر وقت به رختخواب بروم | 60 |
|  |  |  |  |  | می خواستم معلمم مرا دوست داشته باشد | 61 |
|  |  |  |  |  | به جزییات زیاد اهمیت می دادم | 62 |

**تحلیل بر اساس مولفه های پرسشنامه**

به این ترتیب که ابتدا پرسشنامه­ها را بین جامعه خود تقسیم و پس از تکمیل پرسشنامه­ها داده ها را وارد نرم افزار اس پی اس اس کنید. البته قبل از وارد کردن داده ها شما باید پرسشنامه را در نرم افزار اس پی اس اس تعریف کنید و سپس شروع به وارد کردن داده ها کنید.

چگونگی کار را برای شفافیت بیشتر به صورت مرحله به مرحله توضیح می دهیم

مرحله اول. وارد کردن اطلاعات تمامی سوالات پرسشنامه ( دقت کنید که شما باید بر اساس طیف لیکرت عمل کنید .

مرحله دوم. پس از وارد کردن داده های همه سوالات، سوالات مربوط به هر مولفه را کمپیوت(compute) کنید. مثلا اگر مولفه اول X و سوالات آن 1 تا 7 است شما باید سوالات 1 تا 7 را compute کنید تا مولفه x ایجاد شود.

به همین ترتیب همه مولفه ها را ایجاد کنید و پس از این کار در نهایت شما باید همه مولفه ها که ایجاد کردید را با هم compute کنید تا این بار متغیر اصلی تحقیق به وجود بیاید که به طور مثال متغیر مدیریت دانش یا ... است.

مرحله سوم. حالا شما هم مولفه­ها را به وجود آورده اید و هم متغیر اصلی تحقیق را؛ حالا می توانید از گرینه آنالیز هر آزمونی که می خواهید برای این پرسشنامه( متغیر) بگیرید.

مثلا می توانید آزمون توصیفی( میانگین، انحراف استاندارد، واریانس) یا می توانید آزمون همبستگی را با یک متغیر دیگر بگیرید.

**نمره گذاری پرسشنامه:**

نمره گذاری گزینه ها به این صورت انجام می شود که نمره یک برای کاملا مخالف تا نمره پنج برای کاملا موافق در نظر گرفته می شود و پانزده سوال نیز بطور معکوس نمره گذاری می شود که عبارتند از( عبارات شماره 4،12،26،30،35،40،49 ،52،54 ،55 ،59 و60).

برای محاسبه امتیاز هر زیرمقیاس، نمره تک تک گویه های مربوط به آن زیرمقیاس را با هم جمع کنید. برای محاسبه امتیاز کلی پرسشنامه، نمره همه گویه های پرسشنامه را با هم جمع کنید. دامنه امتیاز این پرسشنامه بین 62 تا 310 خواهد بود. هر چه امتیاز حاصل شده از این پرسشنامه بیشتر باشد، نشان دهنده مناسب بودن سبک زندگی فرد خواهد بود و بالعکس.

**روایی و پایایی پرسشنامه**

**روایی ابزار جمع آوری داده ها**

اعتبار یا روایی با این مسئله سر و کار دارد که یک ابزار اندازه گیری تا چه حد چیزی را اندازه می گیرد که ما فکر می کنیم( سرمد و همکاران،1390). در پروژهش بشیری و همکاران (1386) روایی محتوایی و صوری و ملاکی این پرسشنامه مناسب ارزیابی شده است.

**پایایی ابزارهای جمع آوری داده ها**

قابلیت اعتماد یا پایایی یک ابزار عبارت است از درجه ثبات آن در اندازه گیری هر آنچه اندازه می گیرد یعنی اینکه ابزار اندازه گیری در شرایط یکسان تا چه اندازه نتایج یکسانی به دست می دهد( سرمد و همکاران،1390). ضريب آلفاي كرونباخ محاسبه شده در پژوهش بشیری و همکاران (1386) براي این پرسشنامه بالای 7/0 برآورد شد.

**ضريب پايايي پرسشنامه سبک زندگی**

| **پرسشنامه و ابعاد آن** | **ضريب پايايي (الفای کرونباخ)** |
| --- | --- |
| **فردگرایی اقتصادی** | **72/0** |

**منابع:**

سرمد، زهره، حجازی، الهه و عباس بازرگان (1390). ***روش تحقیق در علوم رفتاری***، انتشارات آگاه.

بشیری، مژگان و همکاران. (1386) بررسی ویژگی های روانسنجی و هنجریابی پرسشنامه شیوه ی زندگی بر روی جوانان 18 تا 40 سال. تازه ها و پژوهش های مشاوره 6(21): 69-92.
