# Supplementary material for: Unravelling pain in diabetic neuropathy patients: Exploring the relationship between perceived pain severity, lifestyle, and coping strategies mediated by self-focused attention and rumination: A cross-sectional study
Source: Heliyon. 2025 Jan 31;11(3):e42397. doi: 10.1016/j.heliyon.2025.e42397 (PMC11848071; doi:10.1016/j.heliyon.2025.e42397)
Supplement: Multimedia component 2 [file mmc2.docx]

| **لطفاً به سوالات زیر پاسخ دهید** | | **نداشتن احساس درد** | **احساس درد خیلی کم** | **احساس درد کم** | **ندرتا احساس درد** | **احساس درد متوسط** | **گاهی احساس درد** | **اغلب احساس درد** | **احساس درد زیاد** | **احساس درد خیلی زیاد** | **درد خیلی شدید** |
| --- | --- | --- | --- | --- | --- | --- | --- | --- | --- | --- | --- |
|  | **درد ضربان دار (تپنده)** |  |  |  |  |  |  |  |  |  |  |
|  | **درد تیر کشیدن** |  |  |  |  |  |  |  |  |  |  |
|  | **درد مانند سوراخ کردن** |  |  |  |  |  |  |  |  |  |  |
|  | **درد برنده** |  |  |  |  |  |  |  |  |  |  |
|  | **درد مانند گرفتگی عضله** |  |  |  |  |  |  |  |  |  |  |
|  | **درد تحلیل برنده خورنده- حالت جویده شدن** |  |  |  |  |  |  |  |  |  |  |
|  | **درد سوزنده** |  |  |  |  |  |  |  |  |  |  |
|  | **درد مبهم که منجر به رنج مداوم میشود** |  |  |  |  |  |  |  |  |  |  |
|  | **درد سنگین** |  |  |  |  |  |  |  |  |  |  |
|  | **درد شکننده** |  |  |  |  |  |  |  |  |  |  |
|  | **درد شکافنده** |  |  |  |  |  |  |  |  |  |  |
|  | **درد کسل کننده، درد جانکاه** |  |  |  |  |  |  |  |  |  |  |
|  | **درد تهوع آور** |  |  |  |  |  |  |  |  |  |  |
|  | **درد ترس آور** |  |  |  |  |  |  |  |  |  |  |
|  | **درد زجرآور** |  |  |  |  |  |  |  |  |  |  |
|  | **درد مانند شوک الکتریکی، مثل برق گرفتگی** |  |  |  |  |  |  |  |  |  |  |
|  | **درد با احساس یخ زدگی** |  |  |  |  |  |  |  |  |  |  |
|  | **درد با حالت سیخ فروکردن** |  |  |  |  |  |  |  |  |  |  |
|  | **درد ناشی از لمس سطحی** |  |  |  |  |  |  |  |  |  |  |
|  | **خارش** |  |  |  |  |  |  |  |  |  |  |
|  | **احساس سوزش و سوزن سوزن شدن** |  |  |  |  |  |  |  |  |  |  |
|  | **بی حسی** |  |  |  |  |  |  |  |  |  |  |

اين پرسشنامه 22 مجموعه عبارت داشته و هدف سنجش درک افراد از درد در 1 بعد مي باشد (ملزاک، 1997).
