# Supplementary material for: Unravelling pain in diabetic neuropathy patients: Exploring the relationship between perceived pain severity, lifestyle, and coping strategies mediated by self-focused attention and rumination: A cross-sectional study
Source: Heliyon. 2025 Jan 31;11(3):e42397. doi: 10.1016/j.heliyon.2025.e42397 (PMC11848071; doi:10.1016/j.heliyon.2025.e42397)
Supplement: Multimedia component 3 [file mmc3.docx]

**پرسشنامه راهبردهای مقابله با درد**

**هدف**: ارزیابی انواع راهبردهای مقابله با درد (توجه برگردانی، تفسیر مجدد درد، گفتگو با خود، نادیده انگاشت درد، فاجعه آفرینی و دعا – امیدواری)

***********************************

**شیوه نمره گذاری**

این پرسشنامه دارای 42 سوال بوده و هدف آن ارزیابی انواع راهبردهای مقابله با درد (توجه برگردانی، تفسیر مجدد درد، گفتگو با خود، نادیده انگاشت درد، فاجعه آفرینی و دعا – امیدواری) می باشد. از آزمودنیخواسته می شود تا هر عبارت را خوانده و با استفاده از یک مقیاس 7 درجه ای (صفر تا شش) مشخص نماید که هنگام مواجهه با درد تا چه میزان از هر یک از راهبردهای مذکور استفاده کرده است.

این پرسشنامه دارای 6 بعد می باشد که در جدول زیر ابعاد و نیز شماره سوالات مربوط به هر بعد ارائه گردیده است:

| **بعد** | **سوالات مربوطه** |
| --- | --- |
| توجه برگردانی | 6-1 |
| تفسیر مجدد درد | 12-7 |
| گفتگو با خود | 18-13 |
| نادیده انگاشت درد | 24-19 |
| فاجعه آفرینی | 30-25 |
| دعا – امیدواری | 36-31 |

با استفاده از 2 سوال آخر می توان فهمید که بکار بستن این این راهبردها تا چه حد توانسته است در مقابله با درد موثر بوده باشد. هر بعد نمره ای از 0 تا 36 خواهد داشت. امتیازات بالاتر در هر بعد نشان دهنده آن است که پاسخ دهنده از آن راهبرد بیشتر در مقابله با درد استفاده می نماید. از سوال 37 تا 42 در هیچ کدام از ابعاد جایی ندارند و به بررسی توانایی فعالیت با وجود درد می پردازند.

**روایی و پایایی**

در پژوهش روزنستیل و کیفی (1983) روایی محتوایی با استفاده از خبرگان این علم تایید شده است. روایی سازه نیز با استفاده از تحلیل عاملی در یک عامل به اثبات رسید. برای محاسبه پایایی آن از آلفای کرونباخ استفاده گردید که میزان آن برای تمامی ابعاد بالای 70/0گزارش شد.

***********************************

**Reference:**

***********************************
