# Supplementary material for: Unravelling pain in diabetic neuropathy patients: Exploring the relationship between perceived pain severity, lifestyle, and coping strategies mediated by self-focused attention and rumination: A cross-sectional study
Source: Heliyon. 2025 Jan 31;11(3):e42397. doi: 10.1016/j.heliyon.2025.e42397 (PMC11848071; doi:10.1016/j.heliyon.2025.e42397)
Supplement: Multimedia component 4 [file mmc4.doc]

**پرسشنامه ی نشخواری فکری نالن هوكسما و مارو 1991**

پرسشنامه نشخوار فکری توسط هوکسما و مارو در سال (1991) برای سنجش نشخوار فکری طراحی و تدوین شده است. این پرسشنامه دارای 22 سوال  می باشد و بر اساس طیف چهار گزینه ای لیکرت با سوالاتی مانند (سعی می کنید شخصیت خود را بررسی کنید تا بفهمید که چرا افسرده اید) به سنجش نشخوار فکری می پردازد.

- **مولفه های پرسشنامه**

بروز دادن(بازتاب)[[1]](#footnote-2): 7، 11، 12، 20 و 21

در فکر فرو رفتن[[2]](#footnote-3): 5، 10، 13، 15 و 16

افسردگی[[3]](#footnote-4): 1 تا 4، 6، 8، 9، 14، 17، 18، 19 و 22

|  | **پرسشنامه ی نشخواری فکری نالن هوكسما و مارو 1991** | هرگز | گاهی اوقات | اغلب اوقات | همیشه |
| --- | --- | --- | --- | --- | --- |
| 1- | فکر میکنید که چقدر احساس تنهایی می کنید |  |  |  |  |
| 2- | فکر می کنید اگر در همین حالت باقی بمانید نمی توانید وظایفتان را انجام دهید |  |  |  |  |
| 3- | فکر می کنید احساس دردمندی و خستگی می کنید |  |  |  |  |
| 4- | فکر می کنید که چقدر تمرکز کردن دشوار است |  |  |  |  |
| 5- | فکر میکنید که من چکار کرده ام که به این حالت دچار شده ام |  |  |  |  |
| 6- | فکر می کنید چقدر بی حوصله و بی انگیزه اید |  |  |  |  |
| 7- | با بررسی واقع اخیر سعی می کنید بفهمید چرا افسرده اید |  |  |  |  |
| 8- | فکر میکنید که چرا نسبته به همه چیز بی تفاوت شده ام |  |  |  |  |
| 9- | فکر میکنید که چرا دیگر نمی توانید ادامه دهید |  |  |  |  |
| 10- | فکر میکنید که چرا اغلب به مسائل اینگونه واکنش نشان میدهم |  |  |  |  |
| 11- | با خود خلوت کنید تا برای این احساسات خود دلیل پیدا کنید |  |  |  |  |
| 12- | افکارتان را یادداشت و آنها را بررسی کنید |  |  |  |  |
| 13- | به اوضاع اخیر فکر میکنید و که ای کاش اوضاع بهتر از این بود |  |  |  |  |
| 14- | فکر میکنید اگر نتوانید جلوی این احساساتتان را بگیرید دیگر قادر به تمرکز نخواهید بود |  |  |  |  |
| 15- | به این فکر کنید که چرا مشکلاتی دارم که دیگران ندارند |  |  |  |  |
| 16- | فکر میکنید که چرا نمی توانم بهتر از عهده ی اداره کردن امور برآیم |  |  |  |  |
| 17- | فکر میکنید که چقدر غمگین هستید |  |  |  |  |
| 18- | به همه ی نقاط ضعف ،شکست ها،کاستی ها و اشتباهات خود فکر میکنید |  |  |  |  |
| 19- | فکر میکنید که چرا آنقدر سرحال نیستید که بتوانیدکاری انجام دهید |  |  |  |  |
| 20- | سعی میکنید شخصیت خود را بررسی کنید تا بفهمید که چرا افسرده اید |  |  |  |  |
| 21- | تنهایی به جایی می روید که بتوانید در مورد احساساتتان فکر کنید |  |  |  |  |
| 22- | فکر می کنید که چرا انقدر از دست خودتان عصبانی هستید |  |  |  |  |

**نمره گذاری پرسشنامه:**

| هرگز | گاهی اوقات | اغلب اوقات | همیشه |
| --- | --- | --- | --- |
| 1 | 2 | 3 | 4 |

- **تحلیل ( تفسیر) بر اساس میزان نمره پرسشنامه**

بر اساس این روش از تحلیل شما نمره­های به دست آمده را جمع کرده و سپس بر اساس جدول زیر قضاوت کنید. توجه داشته باشید میزان امتیاز های زیر برای یک پرسشنامه است در صورتی که به طور مثال شما 10 پرسشنامه داشته باشید باید امتیاز های زیر را ضربدر 10 کنید.

مثال: حد پایین نمرات پرسشنامه به طریق زیر بدست آمده است

تعداد سوالات پرسشنامه * 1 = حد پایین نمره

| حد پایین نمره | حد متوسط نمرات | حد بالای نمرات |
| --- | --- | --- |
| 22 | 55 | 88 |

امتیازات خود را از 22 عبارت فوق با یکدیگر جمع نمایید. حداقل امتیاز ممکن 22 و حداکثر 88 خواهد بود.

نمره بین 22 تا 33 : میزان نشخواری فکری پایین است.

نمره بین 33 تا 55 : میزان نشخواری فکری متوسط است.

نمره بالاتر از 55 : میزان نشخواری فکری بالا است.

- **روایی و پایایی:**

نشخوار فکری به عنوان یکی از مهم ترین مولفه ی افسردگی شناخته شده است.از این رو هدف این پژوهش معرفی نشخوار فکری در چارچوب نظریه سبک های پاسخ و پیامد های آن برای خلق غمگین و اختلال افسردگی بود.در این راستا رابطه ی بین نشخوار فکری، افسردگی و اضطراب بر روی نمونه ای از دانشجویان ایرانی مورد بررسی قرار گرفت.شرکت کنندگان 119 نفر و 0.63% زن به روش نمونه گیری تصادفی از دانشجویان دانشگاه فردوسی مشهد انتخاب شدند و پرسشنامه های افسردگی و اضطراب و مقیاس پرسشنامه های نشخواری را کامل کردندضریب آلفای کرونباخ آلفا برابر 0.88 درصد به عنوان شاخصی از همسانی درونی برای مقیاس پاسخ های نشخواری محاسبه شد.نتایج تحلیل رگرسیون سلسه مراتبی نشان داد که نشخوار فکری پس از کنترل اثرات سن ، جنس و میزان اضطراب به طور معنی داری از 30 % از تغییر در واریانس افسردگی را پیش بینی کرد. با وجود تفاوت های فرهنگی و اجتماعی یافته های پیشین در مورد رابطه ی نشخوار فکری و افسردگی در نمونه ی ایرانی نیز تایید شد.به علاوه تایید شد که نشخوار فکری میتواند میزان افسردگی را حتی پس از کنترل میزان اضطراب پیش بینی کنند که این یافته با توجه به نقش نشخوار فکری در اضطراب و علائم مختلط اضطراب– افسردگی حائز اهمیت است.

- **منبع:**

باقری نژاد،مینا،صالحی فدردی .جواد،طباطبایی ، محمود،(1389)،رابطه ی بین نشخوار فکری و افسردگی در نمونه ای از دانشجویان ایرانی ،مطالعات تربیتی و روانشناسی ،11(1)،38-21.

Treynor, W., Gonzalez, R., & Nolen-Hoeksema, S. (2003). Rumination reconsidered: A psychometric analysis. *Cognitive therapy and research*, *27*(3), 247-259.

1. Reflection; [↑](#footnote-ref-2)
2. Brooding; [↑](#footnote-ref-3)
3. Depression-Related [↑](#footnote-ref-4)
