# Supplementary material for: Unravelling pain in diabetic neuropathy patients: Exploring the relationship between perceived pain severity, lifestyle, and coping strategies mediated by self-focused attention and rumination: A cross-sectional study
Source: Heliyon. 2025 Jan 31;11(3):e42397. doi: 10.1016/j.heliyon.2025.e42397 (PMC11848071; doi:10.1016/j.heliyon.2025.e42397)
Supplement: Multimedia component 5 [file mmc5.pdf]

پرسشنامه کانون توجه

ووِدی، چامبلس، گلاس (1997)

لطفاً جملات زیر را به دقت بخوانید و عبارتی را که به بهترین وجه ممکن وضعیت شما را در تعاملات اجتماعی بیان می‌کند با درج علامت × مشخص نمایید.

ردیف	در یک تعامل اجتماعی	اصلاً	کمی	تاحدی	زیاد	کاملاً
۱)	من روی آنچه می‌گویم یا انجام می‌دهم تمرکز می‌کنم.					
۲)	من به تأثیری که روی افراد دیگر می‌گذارم توجه می‌کنم.					
۳)	من روی سطح اضطرابم تمرکز می‌کنم.					
۴)	من روی واکنش‌های بدنی درونی‌ام تمرکز می‌کنم (برای مثال، ضربان قلب).					
۵)	من روی شکست‌های اجتماعی گذشته‌ام تمرکز می‌کنم.					
۶)	من روی ظاهر یا لباس افراد دیگر تمرکز می‌کنم.					
۷)	من روی شرایط یا ویژگی‌های محیط فیزیکی تمرکز می‌کنم.					
۸)	من روی اینکه چگونه افراد دیگر در مورد خودشان احساس می‌کنند تمرکز می‌کنم.					
۹)	من روی آنچه درباره افراد دیگر فکر می‌کنم تمرکز می‌کنم.					
۱۰)	من روی آنچه افراد دیگر می‌گویند یا انجام می‌دهند تمرکز می کنم.					

پرسشنامه کانون توجه[2]: این پرسشنامه به‌وسیله وودی، چامبلس وگلاس (۱۹۹۷) ساخته شده و دارای دو زیر مقیاس با پرسش‌های مدرج پنج نمره‌ای است. زیر مقیاس‌های این پرسشنامه عبارتند از: مقیاس کانون توجه متمرکز برخود (سؤالات ۱تا ۵) و مقیاس کانون توجه بیرونی (سؤالات ۶ تا ۱۰) (چامبلس وگلاس، ۱۹۹۷). آزمودنی‌ها به گویه‌های پرسشنامه بر پایه تصور تعامل اجتماعی پیشین پاسخ می‌دهند. هرگویه شامل یک مقیاس ۵ درجه‌ای است که نشان می‌دهد تاچه اندازه کانون توجه آزمودنی با عبارت‌ها هماهنگ است. نمره‌های هر زیر مقیاس با میانگین پنج گویه مقایسه می‌شود. امتیازات بالاتر در هر بعد نشان‌دهنده تمرکز بیشتر توجه فرد پاسخ‌دهنده در آن بعد از کانون توجه خواهد بود و برعکس. خیر و همکاران (۱۳۸۶) ضریب آلفای کرونباخ را برای زیر مقیاس‌های توجه متمرکز برخود وکانون توجه بیرونی به ترتیب ۰/۷۵ و ۰/۸۶ به دست آورده‌اند. در این پژوهش ضریب آلفای کرونباخ برای عامل کانون توجه متمرکز بر خود (۰/۷۳) و برای عامل توجه بیرونی (۰/۸۲) است.
